# Habitat-Specific Spatiotemporal Patterns of Red Imported Fire Ants in Guangzhou: A Core City of the Guangdong–Hong Kong–Macao Greater Bay Area

**DOI:** 10.3390/insects17040378

**Published:** 2026-04-01

**Authors:** Meng Chen, Yunbo Song, Jingxin Hong, Mingrong Liang, Yuling Liang, Yongyue Lu

**Affiliations:** 1 Red Imported Fire Ant Research Center, South China Agricultural University, Guangzhou 510642, China; cm@stu.scau.edu.cn (M.C.); songyunbo-scau@stu.scau.edu.cn (Y.S.); hongjingxin@stu.scau.edu.cn (J.H.); liangmr0321@connect.hku.hk (M.L.); 2 Insect Biodiversity and Biogeography Laboratory, School of Biological Sciences, The University of Hong Kong, Pok Fu Lam Road, Hong Kong SAR, China

**Keywords:** red imported fire ant, seasonal dynamics, spatial analysis, habitat preference, abandoned farmland, precise management

## Abstract

Understanding the spatiotemporal dynamics and underlying drivers of invasive species is crucial for moving beyond descriptive monitoring to predictive management. The red imported fire ant (*Solenopsis invicta* Buren, RIFA) continues to spread globally, yet studies often lack the seasonal and cross-habitat resolution needed to explain the puzzling heterogeneity of infestations within urban landscapes—such as the stark contrast between high-density agricultural zones and low-density urban green spaces. To address this gap, we conducted a four-season, city-wide survey of 129 sites across four dominant habitat types (farmlands, fishponds, orchards, and urban green spaces) in Guangzhou, a core city of the GBA. Using inverse distance weighting interpolation, kernel density estimation, and spatial autocorrelation, we sought to examine not only the spatial patterns of RIFA distribution but also its potential contributing factors. Our analysis points to three key observations. First, the occurrence level of RIFA appears to follow a significant gradient (farmlands > fishponds > orchards > urban green spaces), suggesting that idle agricultural lands may serve as core reservoirs. Second, we observed a pronounced seasonal bimodal pattern, with peak infestation indices in spring and autumn—a dynamic that seems closely associated with agricultural disturbance cycles. Third, spatial analysis (Global Moran’s I = 0.346, *p* < 0.001) revealed significant clustering, with “high-high” clusters concentrated in peripheral suburban districts. Notably, abandoned or idle farmlands emerged as a potentially important factor, possibly acting as dispersal hubs that help bridge these spatial and temporal peaks and offering one explanation for how local outbreaks may spread across the landscape. Collectively, these findings suggest that RIFA distribution may not be driven solely by static habitat suitability or climate; instead, they point to the importance of considering the dynamic interplay between land-use legacies (such as abandonment), seasonal agricultural practices, and spatial connectivity. By elucidating these drivers, this study refines the theoretical framework of urban invasion biology and provides a replicable, evidence-based control paradigm. We suggest implementing a “zoned, seasonal, and pathway-specific” management strategy that prioritizes suburban farmland complexes during critical seasons and targets abandoned lands for intervention, offering a path towards more sustainable and precise regional RIFA control in the GBA and beyond.

## Simple Summary

The red imported fire ant, a devastating invasive pest, is spreading across China, but effective control has been hampered by a lack of knowledge about where and when they thrive in complex urban landscapes. Most studies look at static snapshots, missing the dynamic “when” and “why”. Over four seasons, we intensively surveyed 129 sites across four habitat types in Guangzhou, a major hub in the Guangdong–Hong Kong–Macao Greater Bay Area (GBA). We discovered that the ants’ distribution is not random: they explode in farmlands and fishponds during spring and autumn, forming concentrated clusters in suburban areas. Crucially, abandoned or idle farmlands may serve as the hidden “distribution centers” fueling this spread. This insight flips the script on control strategies. Instead of blanket spraying, we suggest using a precise, three-pronged approach: target different zones (suburbs vs. city) differently, time your attacks (spring and autumn are critical), and cut off the pathways (abandoned lands are the priority). This study provides city managers with a practical and enforceable management strategy to stop the ants more efficiently, protecting both agricultural production and urban biodiversity.

## 1. Introduction

The red imported fire ant (*Solenopsis invicta*, RIFA) is native to the Parana River Basin in South America, including countries such as Brazil, Paraguay, and Argentina [1]. Since its first detection in Taiwan, China, in 2003 [2,3], and subsequently in Wuchuan, Guangdong Province, in September 2004, this invasive pest has continued to expand its range in mainland China for approximately two decades. The average spread rate of the RIFA in China is approximately 48.1 km/year, with an annual addition of about 26 counties [4,5,6,7,8]. According to the latest data, the RIFA has invaded 703 county-level administrative regions in mainland China, involving 13 provinces and municipalities including Guangdong, Shanghai, and Zhejiang [6,8,9]. The spread mechanism of RIFA involves a complex, multidimensional process encompassing both natural and human-mediated dispersal. Natural dispersal primarily occurs through the mating flights of winged reproductive ants, clustering movements facilitated by buoyancy in water currents, and geographic relocation of ant nests [10,11,12]. These natural events enable RIFA to overcome geographical barriers and colonize new habitats within relatively short timeframes [13,14,15]. Similarly, human factors play a crucial role in the long-distance, cross-regional, and cross-border spread of the RIFA, primarily through the transport and dispersal of soil, plants, turf, waste plastics, and construction debris [6,16,17,18,19].

To investigate the spatial distribution patterns of RIFA, researchers have employed various quantitative methods—including average crowding density, Iwao’s index, clustering indices, clumpiness indices, and negative binomial distribution k-values—to accurately describe their distribution characteristics [20]. Previous studies have shown that RIFA nests exhibit diverse distribution patterns in different habitats. For example, in the agricultural ecosystems of Wuchuan City, Guangdong Province, the nests of RIFAs exhibit a random distribution in the two-dimensional space. In newly invaded green belts, the active nests of RIFAs transition from a uniform distribution to a clustered distribution, while in meadow habitats, they transition from a uniform distribution to a random distribution. Furthermore, across multiple habitats, active nests generally exhibit clustered distribution, whereas in wastelands and field ridges, they tend toward uniform or random patterns [20,21,22,23].

At present, a variety of quantitative approaches have been adopted to analyze the spatial distribution characteristics of RIFA. Among these, geostatistical methods provide crucial technical support for accurately depicting spatial variation in ant nest density and quantifying distributional differences across habitats. For instance, the construction of a spherical model based on multiple lag distances and semivariance values can effectively verify the spatial heterogeneity of ant nest density in different habitats [24,25,26]. Models such as CLIMEX and GARP, combined with climatic factors, have been widely applied to predict the potential suitable habitats and invasion probabilities of RIFAs [27,28,29,30]. Ecological niche models have further confirmed that climatic factors are the core drivers of the geographical distribution of RIFAs [31,32]. In addition, kernel density estimation, spatial autocorrelation models, and machine learning methods can not only accurately capture habitat-specific distribution patterns but also decompose the comprehensive effects of environmental, socioeconomic, and anthropogenic factors (e.g., nighttime light, urban accessibility) on infestation severity—providing a multidimensional perspective for analyzing the drivers underlying habitat preference differences [33,34].

Despite the establishment of an increasingly refined methodological framework in existing studies, notable research gaps remain when examined against monitoring data from different habitats in South China. Most studies have focused on static spatial distribution characteristics, which fail to capture the dynamically fluctuating seasonal patterns of RIFA nest density observed in the field. Furthermore, systematic research on dispersal pathways, dynamic evolutionary processes, and cross-habitat driving mechanisms remains unclear. The Guangdong–Hong Kong–Macao Greater Bay Area (GBA), as a major entry point for international trade and a region undergoing rapid urbanization alongside intensive agriculture, represents a high-risk corridor for RIFA establishment and spread [35]. As a core city of the GBA, Guangzhou shares highly similar habitat types, climatic characteristics, and urbanization processes with other GBA cities. To address these gaps, the present study takes RIFA populations across multiple habitats in Guangzhou as the research object and systematically analyzes their spatial distribution characteristics and habitat adaptability rules by integrating occurrence dynamics and population density data obtained from year-round seasonal monitoring. By synthesizing multi-source analytical methods, this study aims to deepen the understanding of how habitat types, climatic factors, and anthropogenic activities synergistically regulate the spatiotemporal dynamic patterns of RIFA—thereby bridging the existing disconnect between static distribution characterization and dynamic evolutionary analysis.

The objectives of this study are twofold: first, to clarify the core driving mechanisms underlying the spatial distribution of RIFA in Guangzhou and refine the theoretical framework of habitat adaptability for invasive pests; and second, to provide a scientific basis for formulating differentiated prevention and control strategies targeting high-incidence areas (e.g., farmlands and fishponds) versus low-incidence areas (e.g., urban green spaces). Therefore, its monitoring data and research conclusions can serve as an important reference for RIFA survey, risk assessment, and control efforts across the region, providing a replicable and scalable prevention paradigm that enhances the precision, coordination, and sustainability of comprehensive RIFA management throughout the GBA. Additionally, understanding the spatiotemporal dynamics within this specific geopolitical and ecological context is crucial for developing targeted surveillance and control measures in similar subtropical trade hubs globally.

## 2. Materials and Methods

### 2.1. Regional Overview

**Figure 1:**
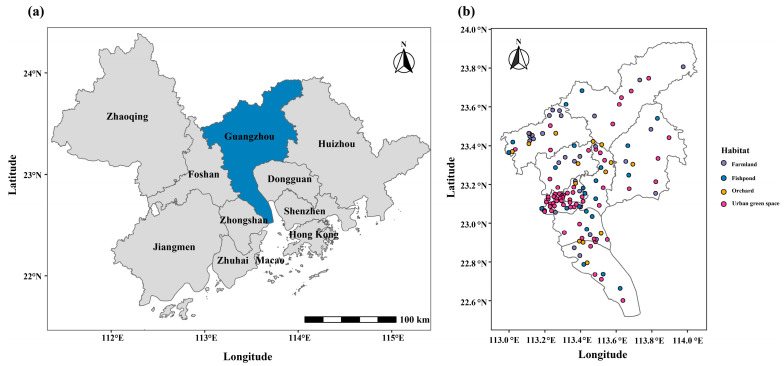
(**a**) Location of Guangzhou within the Guangdong–Hong Kong–Macao Greater Bay Area (GBA); (**b**) distribution of 129 investigation sites across four habitat types (farmlands, fishponds, orchards, and urban green spaces) in Guangzhou.

The study area is situated in Guangzhou (22°26′–23.16′ N, 113°57′–114.3′ E), the capital city of Guangdong Province, China, and a core city of the Guangdong–Hong Kong–Macao Greater Bay Area (GBA)—a major economic zone and international trade hub encompassing Guangzhou, Hong Kong, Macao, and other cities in the Pearl River Delta. Guangzhou serves as a vital transportation hub, connecting various regions both within and beyond the province (Figure 1a). The city experiences a subtropical monsoon climate, characterized by high temperatures and ample precipitation throughout the year. These geographical and climatic conditions, combined with intensive agricultural activities and international trade linkages, create a high-risk environment for the introduction, survival, and proliferation of RIFA.

### 2.2. Survey on the Spatiotemporal Occurrence of RIFA

#### 2.2.1. Habitat Classification and Site Selection

To capture the full spectrum of land-use types potentially affected by RIFA, we classified habitats into four categories based on dominant land-use practices and disturbance regimes, which represent distinct management units for municipal pest control:

Farmlands: Open-field crops subject to annual tillage and frequent anthropogenic disturbance (e.g., rice, vegetables, peanuts, sweet potatoes, maize).

Fishponds: Aquatic-adjacent areas with earthen dykes, characterized by stable moisture and moderate disturbance (e.g., pond embankments and surrounding margins).

Orchards: Perennial tree-crop systems with relatively stable soil structure and partial canopy cover (e.g., guava, lychee, banana plantations).

Urban green spaces: Managed vegetated areas within the urban matrix, subject to regular maintenance such as mowing and pesticide application (e.g., residential areas, parks, streets, school campuses, highway verges, wetlands, and nature reserves).

**Figure 2:**
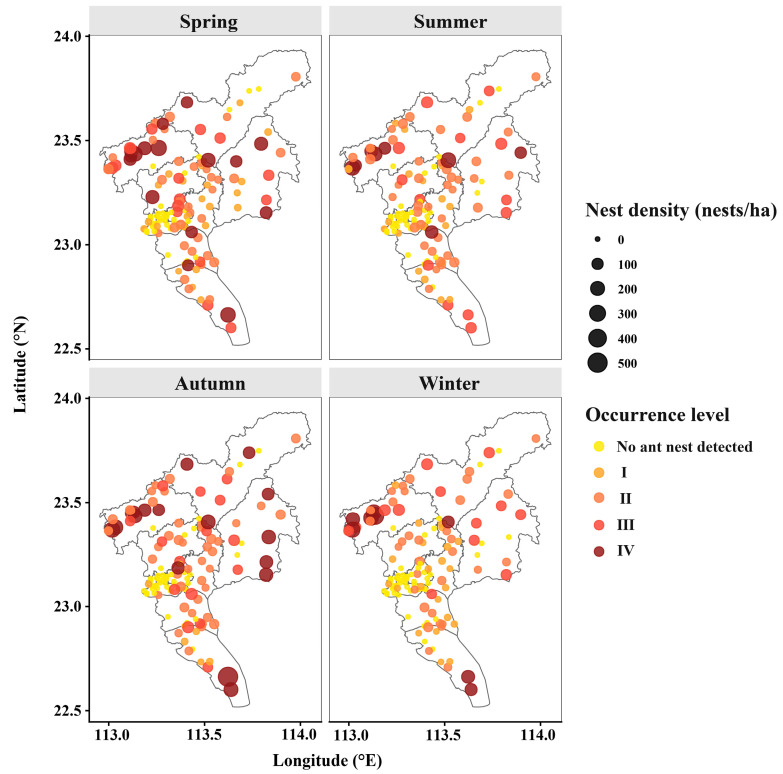
Seasonal spatial distribution and nest density of RIFA. Each point represents a fixed investigation site. Point size indicates nest density (nests ha^−1^), and color denotes occurrence category, including no ant nest was detected, Level I, Level II, Level III, and Level IV. Panels correspond to spring, summer, autumn, and winter surveys conducted in 2023. This figure provides a spatial overview of raw survey data underlying the kernel density and spatial autocorrelation analyses presented in the main text.

To ensure representative spatial coverage across Guangzhou, we employed a semi-randomized site selection strategy. Using Google Earth satellite imagery, we identified potential green spaces and accessible rural areas across all 11 administrative districts. We then applied a grid-based stratification approach: each district was divided into sectors, and candidate survey points were selected with a minimum predefined interval (approximately 2–3 km) to ensure spatial dispersion and reduce autocorrelation bias. Within each sector, specific sampling sites were randomly chosen from the accessible areas identified on the map. A total of 129 investigation sites were selected for seasonal surveys, comprising 63 urban green spaces, 15 orchards, 28 farmlands, and 23 fishponds (Figure 1b and Figure 2). Each survey point covered a minimum area of 1200 m^2^ to ensure representativeness.

#### 2.2.2. Field Survey Protocol

All surveys were conducted by trained personnel on clear days between 9:00–11:00 a.m. and 2:00–5:00 p.m. to maintain consistent environmental conditions and optimize ant activity detection. Seasonal surveys were carried out in the middle of each season from January to December 2023 (spring: average 23 °C; summer: 31 °C; autumn: 24 °C; winter: 18 °C).

Upon arriving at a pre-selected coordinate, investigators performed a systematic meandering search covering the entire accessible area of the site (≥1200 m^2^). This method involved walking in a zigzag pattern to ensure comprehensive coverage rather than following a fixed transect line, thereby minimizing detection bias toward visually obvious mounds. All active RIFA nests (characterized by the presence of worker ants and typical loose soil mounds) were counted and recorded [36]. Survey approach and levels of infestation were referred to the Chinese national standard GB/T 23626-2009 based on the density of live nests [37,38]. Formula for the nest density (*D*) of the RIFA is as follows:

(1) $$D=\frac{N}{S}$$

where *S* represents the surveyed area (in hectares) and *N* represents the number of active RIFA nests.

#### 2.2.3. Handling of Restricted-Access Sites

In cases where a pre-selected sampling point fell on private property or was inaccessible (e.g., fenced-off farmlands or factories), we implemented a replacement protocol: the survey point was shifted to the nearest accessible location with comparable habitat characteristics (e.g., the edge of the same farmland parcel or an adjacent roadside verge with similar vegetation structure). The habitat type was then reclassified based on the actual land use at the surveyed location. This pragmatic approach allowed us to maintain sample size and spatial representation while minimizing bias toward fully public lands.

#### 2.2.4. Fixed-Point Monthly Monitoring in Key Habitats

To complement the broad-scale seasonal surveys and to capture finer-scale temporal dynamics, we established fixed monitoring plots in two habitat types that experience relatively lower anthropogenic disturbance: fishponds and orchards. These habitats serve as important reference sites for understanding natural population fluctuations.

Fishpond site: One representative fishpond was selected in Guangzhou (113.0305088° E, 23.46419113° N), with three permanent plots (each approximately 400 m^2^) established along the pond dykes.

Orchard sites: Three orchards (custard apple, guava, and tea-oil camellia) were selected in Guangzhou (113.0382979° E, 23.46590357° N; 113.018333° E, 23.369011° N), with three permanent plots (each approximately 400 m^2^) established in each orchard.

Monthly surveys were conducted at the end of each month throughout 2023, resulting in 12 surveys per plot. In the tea-oil camellia orchard, we additionally recorded the volume dynamics of individual ant nests to track colony growth and decline patterns. Detailed results from these monthly observations are provided in Appendix A.

### 2.3. Methods for Studying Spatial Patterns of RIFA Occurrence

Systematic data collection was conducted at each survey point, with the precise location of each point recorded using a Global Positioning System (GPS). The number and distribution of RIFA nests were documented. The collected data will be analyzed statistically and spatially to reveal the characteristics and patterns of the spatial distribution of the RIFA occurrence. Further analysis will apply geographic information systems (GIS) and spatial statistical analysis methods to assess the spatial distribution of the extent of the RIFA occurrence. By combining nest data with geospatial information, spatial interpolation analysis (using Inverse Distance Weighting, IDW), kernel density analysis, and spatial autocorrelation analysis were employed.

In the spatial interpolation analysis, the Inverse Distance Weighting (IDW) method was used. This method estimates the density of RIFA occurrence at unobserved locations based on the distance between the point to be measured and the observed points as a weighting factor. The formula is as follows:

(2) $$Z\left(x\right)=\sum_{i=1}^{n}W_{i}Z\left(x_{i}\right)\;$$

In the formula, *Z*(*x*) is the estimated value of the point to be interpolated, *Z*(*x_i_*) is the value of the *i*th interpolation point, n is the number of interpolation points involved, and *Wi* is the weight of the *i*th interpolation point

(3) $$W_{t}=\frac{d_{\left(x,xi\right)}^{-a}}{\sum_{i=1}^{n}d_{\left(x,xi\right)}^{-a}}$$

In the formula, *a* is the distance index value, and *d*(*x*, *xi*) is the distance between the interpolation point *xi* and the target point *x*. The weight *Wi* decreases as distance increases. Using an appropriate distance index value yields a prediction closer to the true value.

Kernel density analysis was referred to the method of Chen et al. (2023) [33] for assessing the spatial distribution density of RIFA nests and identifying high prevalence areas. The formula for kernel density analysis is:

(4) $$f\left(x,y\right)=\frac{1}{t^{2}}\sum_{i=1}^{n}\left\lfloor \frac{3}{\pi }\rho_{i}{\left(1-{\left(\frac{d_{i}}{t}\right)}^{2}\right)}^{2}\right\rfloor$$

In the formula, *f*(*x*, *y*) is the kernel density estimate of the distribution of RIFAs at location (*x*, *y*). n represents the number of RIFA infested plots, *d_i_* is the distance between location (*x*, *y*) and the centroid of the *i*th infested plot, *ρ_i_* is the nest density of the *i*th infested plot, and *t* is the smoothing parameter for the kernel density estimation search radius.

In spatial autocorrelation analysis, the Moran’s Index was used to determine the spatial dependence or patterns of RIFA nest distribution in the study area, identifying clustering or dispersal trends [33]. The Moran’s Index is divided into global Moran’s Index (*I*) and local Moran’s Index (*I*).

The global Moran’s Index (*I*) is used to assess the presence of clustering characteristics in the overall occurrence of RIFAs in the study area. The closer the absolute value of *I* is to 1, the more pronounced the spatial correlation of RIFA occurrence. The formula is as follows:

(5) $$I=\frac{n}{\sum_{i-1}^{n}\sum_{j-1}^{n}W_{ij}}\times \frac{\sum_{i-1}^{n}\sum_{j-1}^{n}W_{ij}\left(\rho_{i}-\bar{\rho }\right)\left(\rho_{j}-\bar{\rho }\right)}{{\sum_{i-1}^{n}\left(\rho_{i}-\bar{\rho }\right)}^{2}}\left(i\neq j\right)$$

The local Moran’s Index (*I_i_*) indicates the significance of clustering between the occurrence level of RIFAs in a plot and adjacent infested plots. Its formula is as follows:

(6) $$I_{i}=\frac{n\left(\rho_{i}-\bar{\rho }\right)\sum_{j-1}^{n}\left(\rho_{j}-\bar{\rho }\right)}{{\sum_{i-1}^{n}\left(\rho_{i}-\bar{\rho }\right)}^{2}}\left(i\neq j\right)$$

In the formula, n is the number of RIFA-infested plots, *ρ_i_* and *ρ_j_* represent the occurrence density of RIFAs in plots *i* and *j*, respectively. $\bar{\rho }$ is the mean density across all infested plots. *W_ij_* is the spatial weight matrix.

The calculation formula for the mound volume of RIFAs is as follows:

(7) $$V=\frac{2}{3}\pi abc\;$$

In the formula, The basal area of the mound *S* = *πab*, where *a* is half the length of the mound, *b* is half the width of the mound, and *c* is the height of the mound [39].

### 2.4. Data Analysis

Data entry and compilation were performed in Microsoft Excel 2013. Data processing, statistical analyses, and figure generation were conducted using both Python (Version 3.14) and R (Version 4.5). Spatial analyses, Spatial analyses, including inverse distance weighting (IDW) interpolation, kernel density estimation, and spatial autocorrelation analysis (global and local Moran’s I), were implemented in Python using relevant libraries. ArcMap (Version 10.8.2) was used for cartographic visualization and layout of spatial outputs.

**Figure A1:**
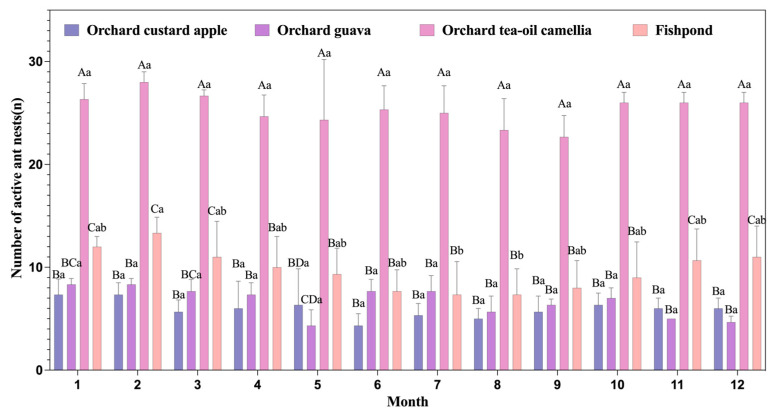
Monthly dynamic changes in the number of RIFA nests in four habitats (orchard custard apple, orchard guava, orchard tea-oil camellia, and fishpond). Nest number was analyzed using two-way ANOVA with month and habitat as fixed factors, followed by Tukey’s multiple comparisons test. Uppercase letters indicate comparisons between habitats within the same month, and lowercase letters indicate comparisons among months within the same habitat; values sharing the same letter are not significantly different (*p* ≥ 0.05).

Season- and habitat-related differences in RIFA nest abundance and nest density were analysed in R. Because the response variables deviated from normality, habitat differences within each season were tested using Kruskal–Wallis tests, followed by Dunn’s post hoc pairwise comparisons among the four habitats. To account for multiple pairwise comparisons within a season, *p* values from Dunn’s tests were adjusted using the Benjamini–Hochberg procedure to control the false discovery rate (FDR), and BH-adjusted *p* values (*p_adj_*) were used for statistical inference. Compact letter displays were generated to summarize significant pairwise differences and were used for figure annotation. Monthly dynamics analyses reported in Appendix A (Figure A1) were performed in GraphPad Prism (version 10.2.0). For these monthly datasets, we used ordinary two-way ANOVA with month and habitat as fixed factors, followed by Tukey’s multiple comparisons test for post hoc contrasts. Model assumptions were evaluated using standard residual diagnostics in Prism; where required, appropriate transformations were considered.

## 3. Results

### 3.1. Occurrence Dynamics and Density of RIFAs Across Habitats

**Figure 3:**
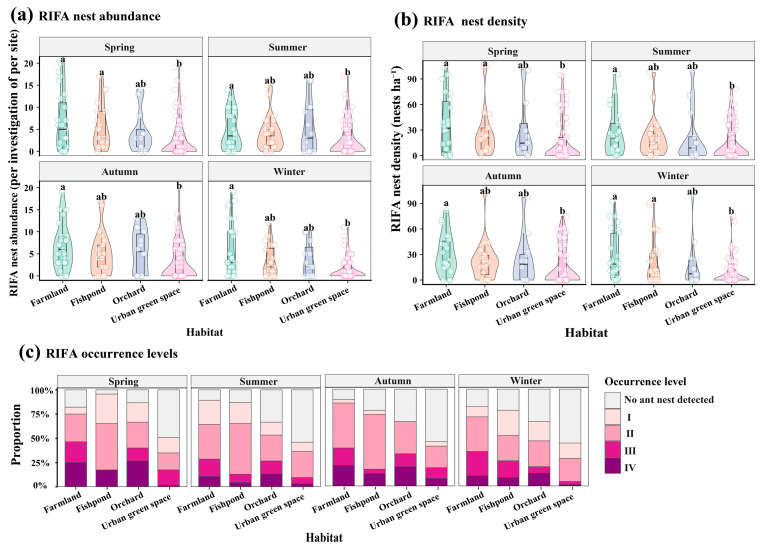
Seasonal variation in abundance, density, and occurrence levels of RIFA across habitats in Guangzhou. (**a**) RIFA nest abundance (number of nests per investigation site) across farmland, fishpond, orchard, and urban green space in spring, summer, autumn, and winter. (**b**) RIFA nest density (nests ha^−1^) across habitats and seasons. (**c**) RIFA occurrence level composition, showing the proportional distribution of occurrence levels within each habitat and season, including that no ant nest was detected, and Levels I–IV represent increasing occurrence intensity. In panels (**a**,**b**), violin plots illustrate the distribution of observations, with overlaid boxplots indicating medians and interquartile ranges and jittered points representing individual survey sites. Different lowercase letters (**a**,**b**) above violins indicate significant differences among habitats within the same season, as determined by Kruskal–Wallis tests followed by Dunn’s post hoc comparisons with Benjamini–Hochberg correction (*p_adj_* < 0.05); habitats sharing the same letter do not differ significantly. In panel (**c**), stacked bars represent the proportional contribution of each occurrence level within habitat–season combinations.

A total of 2825 nests were identified, covering a cumulative area of 69.22 hectares. Overall, Class I and Class II infestations dominated in Guangzhou, with infested areas accounting for 71.11% in spring, 94.41% in summer, 68.03% in fall, and 79% in winter. Specially, in spring, *S. invicta* nest abundance differed significantly among habitats (Kruskal–Wallis test: χ^2^ = 12.54, df = 3, *p* = 0.0057; Figure 3a). Mean nest abundance was highest in farmland (10.6 ± 2.06 nests per site), followed by orchard (8.60 ± 2.45) and fishpond habitats (8.09 ± 2.26), whereas urban green land showed substantially lower abundance (3.30 ± 0.63). Dunn tests indicated that urban green land supported significantly fewer nests than farmland and fishpond habitats (*p_adj_* ≈ 0.018), while differences among farmland, fishpond, and orchard were not significant. In summer, nest abundance also differed significantly among habitats (Kruskal–Wallis test: χ^2^ = 11.89, df = 3, *p* = 0.0078). Farmland exhibited the highest mean nest abundance (6.57 ± 1.49), followed by orchard (5.13 ± 1.49) and fishpond habitats (4.91 ± 1.12), whereas urban green land again showed the lowest abundance (3.05 ± 0.64). Dunn tests revealed that farmland supported significantly higher nest abundance than urban green land (*padj* < 0.05), with no other significant pairwise differences detected. In autumn, significant habitat-related differences in nest abundance were observed (Kruskal–Wallis test: χ^2^ = 13.87, df = 3, *p* = 0.0031). Mean nest abundance was highest in fishpond habitats (10.6 ± 4.82), followed by farmland (8.75 ± 1.48) and orchard (6.53 ± 1.62), while urban green land remained lower (4.92 ± 1.06). Post hoc comparisons showed that farmland supported significantly higher nest abundance than urban green land (*p_adj_* < 0.05), whereas other pairwise contrasts were not significant. In winter, nest abundance exhibited the strongest habitat differentiation (Kruskal–Wallis test: χ^2^ = 20.07, df = 3, *p* = 0.00016). Mean nest abundance declined across all habitats but remained relatively high in farmland (7.68 ± 2.09) and fishpond habitats (6.78 ± 2.04), followed by orchard (3.80 ± 1.02), and reached the lowest level in urban green land (1.83 ± 0.44). That urban green land supported significantly fewer nests than all other habitat types (*p_adj_* < 0.01), while farmland, fishpond, and orchard did not differ significantly from one another.

Across all four seasons, habitat type exerted a significant influence on *S. invicta* nest density (Figure 3b). Kruskal–Wallis tests revealed significant differences among habitats in spring (χ^2^ = 12.49, df = 3, *p* = 0.0059), summer (χ^2^ = 11.58, df = 3, *p* = 0.0090), autumn (χ^2^ = 9.53, df = 3, *p* = 0.023), and winter (χ^2^ = 15.76, df = 3, *p* = 0.0013), indicating consistent habitat effects on nest density throughout the annual cycle. In spring, mean nest density was highest in farmland (36.7 ± 7.29 nests ha^−1^), followed by orchard (30.3 ± 10.7 nests ha^−1^) and fishpond habitats (27.2 ± 6.40 nests ha^−1^), whereas urban green land exhibited substantially lower density (17.4 ± 3.40 nests ha^−1^). Post hoc Dunn tests showed that farmland and fishpond habitats supported significantly higher nest density than urban green land (*p_adj_* = 0.028 for both comparisons), while orchard did not differ significantly from the other habitats. In summer, nest density remained highest in farmland (26.8 ± 5.27 nests ha^−1^), followed by orchard (21.8 ± 8.88 nests ha^−1^) and fishpond habitats (21.5 ± 4.95 nests ha^−1^), and was lowest in urban green land (14.0 ± 2.69 nests ha^−1^). Farmland maintained significantly higher density than urban green land (*p_adj_* = 0.014), with no other significant pairwise differences detected. In autumn, mean nest density again peaked in farmland (31.0 ± 4.98 nests ha^−1^), followed by orchard (22.8 ± 8.42 nests ha^−1^) and fishpond habitats (22.6 ± 5.32 nests ha^−1^), while urban green land showed lower density (15.9 ± 3.01 nests ha^−1^). Pairwise comparisons indicated that farmland supported significantly higher density than urban green land (*p_adj_* = 0.017), whereas other contrasts were not significant. In winter, habitat-related differentiation in nest density was most pronounced. Mean density declined across all habitats but remained relatively high in farmland (28.0 ± 5.72 nests ha^−1^) and fishpond habitats (21.5 ± 5.60 nests ha^−1^), followed by orchard (17.4 ± 7.80 nests ha^−1^), and reached the lowest level in urban green land (8.90 ± 2.02 nests ha^−1^). Urban green land exhibited significantly lower nest density than farmland (*p_adj_* = 0.0019) and fishpond habitats (*p_adj_* = 0.031), while differences among farmland, fishpond, and orchard were not statistically significant.

Across all four seasons, occurrence level composition showed pronounced habitat-specific patterns (Figure 3c and Table A1). Urban green space was consistently dominated by no ant nest detected, accounting for approximately 49–56% of observations across seasons, whereas farmland and fishpond exhibited substantially lower proportions of no ant nest detected (generally <20%). In contrast, moderate to high levels of occurrence were observed across all four seasons in farmland—particularly abandoned or idle plots—as well as in orchard and fishpond habitats. In spring, farmland areas recorded an average Level III, with some surveyed plots reaching Level IV (see Supplementary Materials). Orchard and fishpond habitats averaged Level II, though certain plots also reached Level IV. Occurrences remained rare in urban green spaces, although a few sites reached Level III and IV. Overall, across habitats, Level II represented the most common occurrence category, accounting for roughly 28–40% of observations depending on season. Despite an overall seasonal decline in activity during winter, habitat-related differentiation in occurrence severity was most pronounced, with Urban green space characterized by frequent non-detection and farmland supporting recurrent high-severity occurrence.

As a supplementary field metric, dynamic changes in the number of RIFA nests were compared in four habitats (tea-oil camellia orchard, fishpond, sugar apple orchard, and guava orchard; Appendix A Figure A1) and monthly mound-volume measurements were obtained for a subset of habitats (tea-oil camellia orchard and fishpond); detailed results are provided in Appendix A Table A2.

### 3.2. Spatial Distribution of RIFA Occurrence in Various Districts of Guangzhou

**Figure 4:**
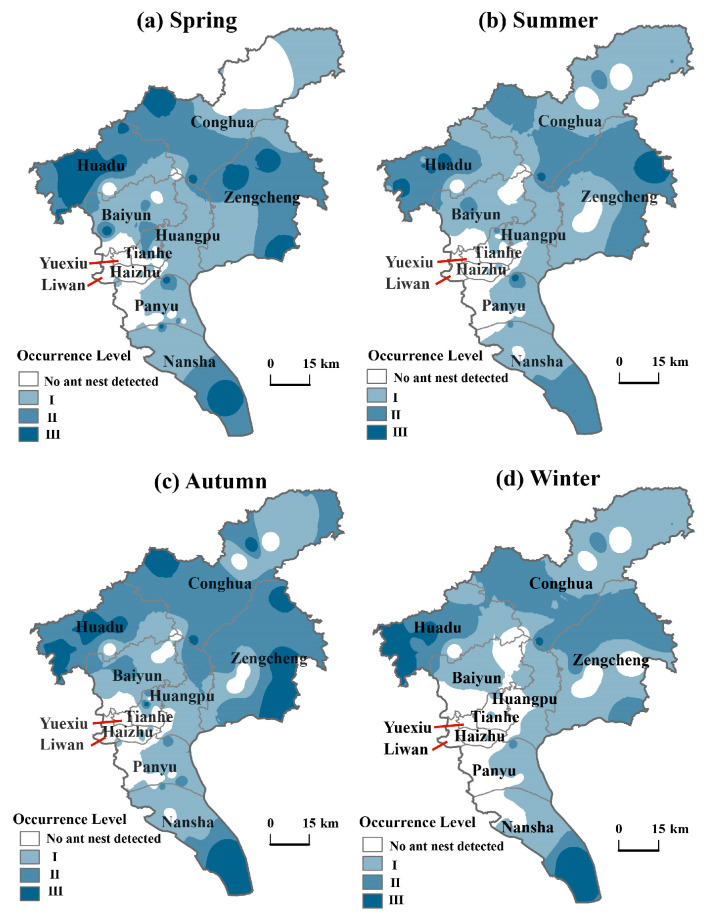
Spatial distribution of RIFA occurrence in Guangzhou. Darker colors represent more severe levels of RIFA occurrence. Panels (**a**), (**b**), (**c**) and (**d**) are for spring, summer, autumn and winter respectively. Areas are classified into four categories based on occurrence severity: no ant nest was detected, Level I, Level II, and Level III, with darker colors indicating higher infestation intensity.

**Figure A2:**
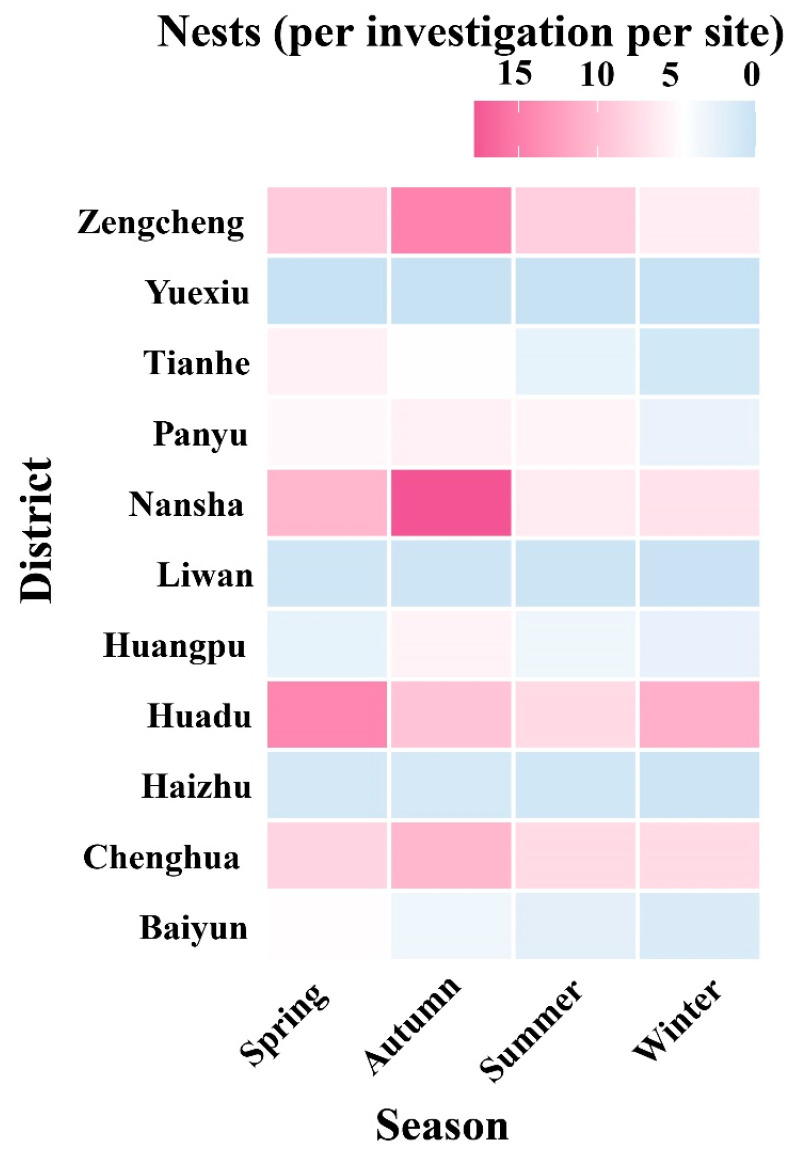
Seasonal dynamics of RIFA nests across 11 districts in Guangzhou. The gradient colors in the heatmap indicate that dark red represents a high number of RIFA nests, transitioning to blue where no RIFA nests were detected.

The severity of RIFA infestations in Guangzhou varied significantly across seasons, with distinct spatial distribution patterns observed in different regions during different seasons (Figure 4 and Appendix A Figure A2).

Survey results indicate that the severity is highest in autumn, followed by spring, winter, and summer. In spring and autumn, areas with severe (Level III) infestations were mainly concentrated in the Huadu, Conghua, Zengcheng, and Nansha districts. During summer, Level III infestations were relatively sparse and scattered, with isolated occurrences in Huadu and Zengcheng districts. In winter, these infestations were primarily concentrated in Huadu and Nansha districts. Overall, Level III infestations exhibited a dispersed distribution pattern, while Level I and II infestations were more concentrated. The spatial distribution of RIFA infestations in Guangzhou tends to radiate outward from the central Yuexiu District, with increasing severity as it spreads.

### 3.3. Spatial Kernel Density Analysis of RIFA Distribution in Guangzhou Districts

**Figure 5:**
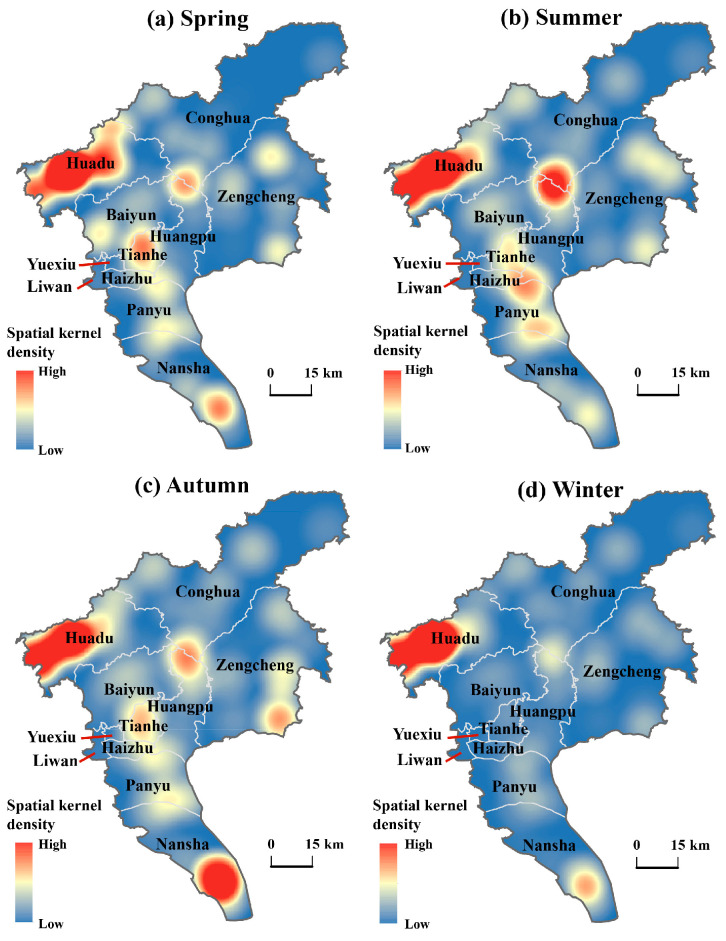
Spatial kernel density map of RIFA occurrence level in Guangzhou area. Darker red areas represent higher densities of RIFAs and blue areas represent lower densities. Panels (**a**), (**b**), (**c**) and (**d**) are for spring, summer, autumn and winter.

The kernel density analysis indicated that the spatial distribution of RIFA infestations in Guangzhou exhibits distinct patterns of localized clustering (Figure 5). High-density clusters were consistently observed in Huadu District across all four seasons. Other areas with high clustering were mainly located at boundary regions, including the borders between Baiyun and Tianhe Districts, Conghua and Huangpu Districts, Haizhu and Panyu Districts, and Nansha District. In contrast, Liwan, Yuexiu, and Haizhu Districts show low levels of clustering. The spatial kernel density of RIFA infestations was more localized and concentrated in spring, summer, and autumn compared to winter. Overall, the seasonal distribution pattern in Guangzhou was characterized by “localized high clustering and widespread dispersion”.

### 3.4. Spatial Autocorrelation Analysis of RIFA Infested Areas in Guangzhou Districts

The calculated global Moran’s *I* index for RIFA infestation areas averaged 0.34611, with a Z-score of 11.3041 and a *p*-value of less than 0.001, indicating statistical significance (Table 1). The results suggest that the severity of RIFA infestations in Guangzhou exhibits a positive spatial autocorrelation.

**Figure 6:**
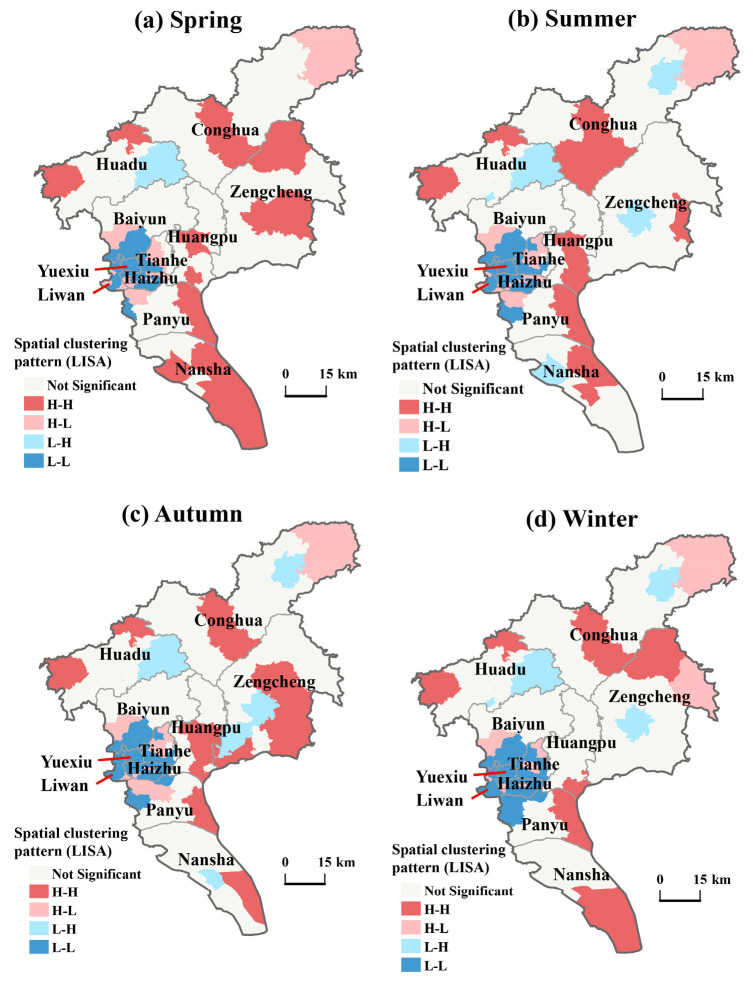
Spatial autocorrelation (LISA) of RIFA occurrence level in Guangzhou, China. Note: “not significant” indicates areas without significant local spatial autocorrelation. H–H and L–L denote significant positive local spatial autocorrelation (high–high and low–low clusters), whereas H–L and L–H represent negative local spatial autocorrelation (spatial outliers: high surrounded by low, or low surrounded by high). Panels (**a**–**d**) correspond to spring, summer, autumn, and winter, respectively. LISA cluster maps were generated using Local Moran’s *I* with a significance threshold of *p* < 0.05.

Using the local Moran’s I index and Local Indicators of Spatial Association (LISA) maps, the local clustering characteristics of RIFA infestations in Guangzhou were further analyzed and spatially visualized (Figure 6). The analysis indicates that high-high clustering areas are primarily concentrated in the outer districts of Guangzhou. In spring, these high-high clustering areas are mainly found in Nansha and Zengcheng Districts, with some occurrences in Huadu, Conghua, Huangpu, and Panyu Districts. In summer, they are primarily concentrated in Conghua District; in autumn, mainly in Zengcheng District; and in winter, in parts of Huadu, Conghua, Zengcheng, Huangpu, and Panyu Districts. Additionally, high-low and low-high clustering areas are mostly distributed in the surrounding areas of Conghua, Huadu, and Yuexiu Districts, showing a scattered pattern. In contrast, Yuexiu, Tianhe, Liwan, and Haizhu Districts consistently exhibit low-low clustering throughout the year. Furthermore, certain parts of Panyu District also display low-low clustering characteristics.

## 4. Discussion

RIFAs are thermophilic, hygrophilous, omnivorous social insects with extremely strong adaptability and aggressiveness, capable of reproducing and spreading in various habitats in invaded areas [4]. Our study revealed a habitat gradient in the annual average density of RIFAs in Guangzhou: farmlands > fishponds > orchards > urban green spaces, which is highly consistent with the findings of Chen et al. (2023) [33] on Haitan Island, Fujian Province. Both studies suggest that farmlands may serve as the most preferred habitat for RIFAs, potentially due to a combination of sustained resource inputs—such as crops, organic fertilizers, and associated arthropods—and suitable nesting substrates [40]. Comparison with surveys in Wenshan (Yunnan) and northern Guangdong [23,41] also suggests that farmlands may consistently harbor the highest RIFA nest densities across regions. This points to a possible pattern in habitat preference within subtropical areas, with farmlands potentially serving as core habitats characterized by high resource availability and stable nesting opportunities across South China. With accelerated urbanization and continuous urban expansion in Guangzhou, some farmlands have been converted to other uses, leading to partial abandonment—which in turn provides undisturbed, ideal habitats for RIFAs [4]. Agricultural habitats are also subject to routine management (e.g., tillage, fertilization, irrigation, and crop-protection interventions), which can reshape ant assemblages and competitive interactions. Evidence from pasture systems indicates that management regimes and disturbance contexts can substantially alter RIFA mound abundance [42]. Farm management may further restructure enemy communities and resource landscapes; for example, increased floral resource diversity can disproportionately favor invasive ants relative to non-invasive natural enemies, potentially reinforcing RIFA dominance [43]. Importantly, targeted chemical control aimed specifically at RIFA (e.g., baits or dust applications) can suppress RIFA populations and is associated with recovery of ant-community diversity [37]. Mechanistically, effective exposure of colony members to pesticides can differ markedly across compounds and application routes, particularly for soil-nesting social insects, reinforcing the need to record pesticide-use metadata to evaluate management effects explicitly in future surveys [44]. However, because standardized pesticide-use information (active ingredients, application frequency, timing, and application method) was unavailable for our farmland sites, we could not quantify pesticide exposure or test its contribution statistically. We therefore interpret the consistently high nest abundance and density observed in farmland as evidence of persistence under common agricultural contexts, while treating pesticide use as a plausible but untested driver that should be prioritized in future monitoring and mechanistic analyses.

Although fishponds can also provide abundant food resources—such as insects, aquatic plants, and even dead fish sometimes discarded by farmers along the embankments (authors’ observation)—the variety and quantity of these resources may be slightly lower than those found in farmlands [21]. Nevertheless, the environment around fishponds also offers relatively suitable conditions for the survival and reproduction of RIFAs [41]. Moreover, to minimize negative impacts of chemical agents on aquatic resources, most farmers rarely use pesticides to control RIFAs in fishpond areas. As urbanization advances, natural and semi-natural habitats decline, making urban green spaces important refuges for biodiversity. However, such green spaces in densely populated and frequently managed environments (e.g., parks and road green belts in old urban areas) are not highly suitable for RIFA survival [33]. Conversely, newly developed urban green spaces—facilitated by vegetation transportation—remain a key pathway for RIFA introduction and spread [45,46].

In this study, a distinct seasonal pattern was observed in Guangzhou, with infestation levels appearing highest in spring and autumn and moderate in summer and winter. Previous studies have confirmed that invasive RIFA populations are more abundant in areas with annual precipitation of 516.4 mm, annual average temperature of 18.6 °C, or altitude of 569.9 m [47]. Internationally, RIFAs in central Texas, USA, also show peak activity in spring [48], consistent with our Guangzhou findings. However, the seasonal dynamics of RIFAs in Guangzhou differ from those in Yiliang, Yunnan Province, where the peak periods of RIFA population size and active mound occurrence fall in summer and autumn [49]. This discrepancy likely reflects climatic differences: Guangzhou’s subtropical climate makes spring and autumn optimal for RIFA reproduction, whereas Yiliang—located in the subtropical-temperate transition zone—experiences favorable conditions in summer and autumn. This comparison aligns with ecological niche model predictions by Song et al. (2021) [50] and Wang et al. (2018) [51], indicating that climatic factors (especially temperature ranges) determine regional patterns of RIFA seasonal dynamics.

Moreover, this study found that RIFA densities in farmlands, fishponds, and orchards remained relatively stable across all four seasons—unlike urban green spaces. This stability likely reflects RIFAs’ strong ecological adaptability, enabling them to maintain population densities despite seasonal changes. Although seasonal fluctuations affect food resources, the food supply in fishponds, farmlands, and orchards remains sufficient to support RIFA survival year-round. Additionally, the presence of congeneric species or food competitors in these habitats may limit RIFA population growth. In contrast, urban green spaces experience high human foot traffic, frequent environmental changes, and significant anthropogenic disturbance, which can reduce worker ant activity and thereby influence mound density [52]. This finding extends the work of Sun (2009) [22], who focused only on seasonal dynamics in a single habitat, and confirms that strong ecological adaptability and sufficient food resources can offset seasonal temperature fluctuations—a dimension not addressed in Xu et al.’s (2010) [21] study of newly invaded areas.

Mound (nest) volume provides complementary information on mound-building investment and near-nest microhabitat conditions. In fire ants, mound volume has been reported to covary with colony biomass and to vary seasonally, broadly tracking changes in colony condition and brood production [53]. However, mound structure is also sensitive to short-term disturbance and environmental context (e.g., rainfall, soil properties, and mowing/maintenance), which can inflate or collapse mound volume without proportional demographic change. Therefore, in the present study we interpret the orchard–fishpond mound-volume dynamics (Appendix A Figure A1; Table A2) as supporting evidence for season-dependent mound construction and maintenance, rather than as a direct proxy for colony size. These results are consistent with habitat-specific evidence from South China showing seasonal dynamics of *S*. *invicta* colony structural metrics across orchards and fishponds [54] and with orchard-scale evidence that arthropod community dynamics can shift markedly under RIFA invasion in guava and cherimoya plantations [55]. Nevertheless, linking mound volume to colony demography in Guangzhou would require repeated measurements coupled with nest excavation or independent colony-size estimates in the future.

Additionally, our study comprehensively applied Inverse Distance Weighting (IDW) interpolation, kernel density analysis, and spatial autocorrelation analysis to systematically examine RIFA infestation in Guangzhou. The results showed that the spatial pattern of RIFAs in Guangzhou was characterized by high local aggregation and extensive global diffusion, with significant positive spatial correlation. This finding aligns with Chen et al. (2023) [33] on Haitan Island, Fujian, where RIFAs exhibited high coastal aggregation and scattered global distribution, with high-high aggregation areas concentrated in suburban regions with low human activity. This indicates that the dual pattern of aggregation-diffusion is a common distribution model for RIFAs in urban ecosystems, driven by the combined effects of anthropogenic diffusion (for global distribution) and habitat adaptability (for local aggregation).

Spatial analysis clearly identified high-high aggregation areas primarily in Guangzhou’s peripheral districts: Huadu, Conghua, Zengcheng, Huangpu, Panyu, and Nansha. High-low and low-high aggregation areas were scattered in boundary zones (e.g., Conghua, Huadu, Yuexiu), while low-low aggregation dominated the central urban core (Yuexiu, Tianhe, Liwan, Haizhu) and parts of Panyu. This core-periphery gradient—”high-high aggregation in peripheral districts, low-low aggregation in the central urban area”—is highly consistent with Tian et al.’s (2025) [56] study in Fujian Province, confirming that urbanization intensity is a key landscape factor regulating RIFA spatial patterns. This cross-city validation enhances the universality of this conclusion.

Concurrently, our results suggest that abandoned or idle farmland may serve as a region-specific dispersal source—a potentially important factor that could act as dispersal hubs (Figure 3c and Table A1), reflecting the urbanization dynamics of Guangzhou. Such idle farmland, along with the wasteland or meadow resulting from rapid urbanization, may provide undisturbed breeding grounds for RIFAs—a mechanism not addressed in early research by Zeng et al. (2005) [4], yet consistent with Ying et al.’s (2021) [17] study, supporting that farmland transformation during urbanization is a unique dispersal driver in South China’s coastal cities. Additionally, this study identified fragmented aggregation at district/county boundaries, consistent with Rayment’s (2006) [18] research on weak cross-regional quarantine zones in Australia. This finding emphasizes the necessity of cross-regional quarantine for blocking boundary dispersal as a prevention and control strategy. Targeted and efficient prevention and control measures should be formulated for the high-aggregation areas of RIFAs in Guangzhou and beyond. Meanwhile, given their overall scattered distribution and frequent occurrence at the boundaries of districts and counties, it is imperative to strengthen cross-regional quarantine efforts to reduce the risk of RIFA dispersal to uninfested areas.

Based on these findings, we suggest implementing a “zoned, seasonal, and pathway-specific” prevention and control strategy. Specifically: (1) high-high aggregation areas—specifically farmlands and fishponds—should be prioritized for intensive nest eradication, with efforts focused particularly in spring and autumn, and extended to peripheral areas during winter; (2) fishpond surroundings should prioritize physical or non-chemical control to avoid pollution risks on aquatic resources, aligning with Nester’s (2007) [57] recommendations for aquatic protection zones; (3) for newly established urban green spaces, vector quarantine measures—such as soil treatment prior to transplanting—are required to reduce introduction risk [17]. Furthermore, we recommend establishing isolation and monitoring zones in high-low aggregation areas (boundary transition zones), consistent with Wang et al. (2023) [58]. This practice directly translates spatial analysis outputs into actionable control measures, achieving seamless integration of research conclusions and management practices.

Although this study offers certain insights and serves as a reference, it has several limitations. First, sampling numbers were uneven across habitat types, and overall habitat coverage remained incomplete. While we included four common habitat types—urban green spaces, orchards, farmlands, and fishponds—we did not cover other potential habitats such as mountainous areas, riverbanks, and abandoned industrial zones. Notably, existing studies suggest that the humid environments near low-disturbance mountain edges may serve as concealed breeding grounds for RIFAs [59,60]. Omission of these habitats may lead to incomplete understanding of Guangzhou’s overall RIFA distribution pattern. We have also recognized that aggregating diverse crop types (e.g., vegetables vs. rice paddies) into a single ‘farmland’ category may obscure finer-scale ecological preferences. For instance, although RIFAs prefer to build their nests on the ridges of paddy fields, irrigation frequency in these farmlands might suppress ant nests compared to drier vegetable plots. Future studies should aim for finer taxonomic resolution of habitat types, potentially incorporating soil moisture and canopy cover as continuous variables to disentangle the drivers within these broad categories.

In the future, we will expand habitat investigation and dynamic monitoring by supplementing sampling sites in mountainous areas, river banks, abandoned industrial zones and other habitats, optimizing spatial uniformity of sampling distribution. A dual monitoring system will be established, comprising static fixed sampling sites and dynamic habitat transformation sampling sites, to track RIFA population density and nest location changes during habitat transformations (e.g., farmland abandonment, new green space construction). Continuous monitoring over 3–5 years will provide interannual dynamic data. Moreover, a more robust spatial analysis integrating environmental covariates would greatly enhance predictive power. While our current dataset provides a robust spatiotemporal baseline, it does not yet model the specific environmental drivers—such as land surface temperature, soil texture, or proximity to plant nurseries (a known introduction pathway)—that likely dictate the observed clustering patterns. A logical next step would be to integrate monthly or seasonally RIFA occurrence data—including GIS information for each nest—with high-resolution spatial layers (such as Sentinel satellite data for vegetation indices and digital elevation models for drainage patterns) within a modeling framework such as Maximum Entropy (MaxEnt) or Random Forest. This would allow us to move from describing where they are to predicting why they are there, thereby increasing confidence in the causal mechanisms suggested by our current work. Simultaneously, RIFA samples will be collected from different high-aggregation areas and boundary zones, and population genetic structure analysis will be performed using mitochondrial DNA or SSR markers to identify dispersal sources and genetic exchange pathways.

## 5. Conclusions

This study provided a multi-habitat, four-season analysis of RIFA spatiotemporal dynamics in Guangzhou, a core city of the Guangdong–Hong Kong–Macao Greater Bay Area. Through surveys conducted across 129 sites encompassing farmlands, fishponds, orchards, and urban green spaces—combined with inverse distance weighting interpolation, kernel density estimation, and spatial autocorrelation—this study identified three fundamental patterns. First, RIFA occurrence generally exhibits a significant and stable gradient: farmlands > fishponds > orchards > urban green spaces. Farmlands and fishponds appear to function as core habitats for RIFA propagation, likely due to low anthropogenic disturbance and abundant food resources, whereas urban green spaces show consistently low infestation levels. Second, seasonal dynamics follow a pattern of high infestation in spring and autumn and moderate infestation in summer and winter, which appears closely linked to the subtropical monsoon climate and agricultural disturbance cycles. Third, spatial distribution is characterized by localized high clustering and widespread dispersion, with significant positive spatial autocorrelation. High-high aggregation clusters are concentrated in peripheral districts (Huadu, Conghua, Zengcheng), while low-low aggregation predominates in the central urban core.

This study also identified urbanization-induced farmland abandonment as a potential region-specific dispersal source that may help bridge the observed spatial and temporal patterns by providing undisturbed breeding grounds that support population outbreaks. This finding suggests a need to move beyond static habitat description toward considering the dynamic interplay between land-use legacies, seasonal agricultural practices, and spatial connectivity as synergistic drivers of RIFA invasion.

For practical management, we suggest implementing a “zoned, seasonal, and pathway-specific” control strategy: (1) prioritize intensive nest eradication in high-high aggregation areas (e.g., farmlands, fishponds) during spring and autumn peaks; (2) establish cross-regional quarantine zones at district boundaries to help block dispersal pathways, and adopt physical or non-chemical control methods around fishponds to protect aquatic resources; and (3) implement vector quarantine for newly established urban green spaces through soil treatment prior to plant transplantation. This framework is intended to serve as a practical reference for Guangzhou, other cities in the GBA, and similar subtropical trade hubs globally, with the goal of enhancing the precision, coordination, and sustainability of RIFA management.

## Figures and Tables

**Table 1:** Global Moran index *I* of RIFA occurrence in Guangzhou.

Seasonality	Moran’s I	Z-Scores	*p*
spring	0.310707	10.748543	<0.001
summer	0.347731	11.289688	<0.001
autumn	0.379900	11.913082	<0.001
winter	0.346271	11.265087	<0.001

Note: Moran’s I > 0 indicates positive spatial autocorrelation, meaning similar values are clustered together. Moran’s I < 0 indicates negative spatial autocorrelation, where dissimilar values are near each other. Moran’s I = 0 indicates spatial randomness, with observations distributed randomly in space. High or low Z-scores (deviating from zero) indicate that the observed spatial patterns are significant and not randomly generated.

## Data Availability

The original contributions presented in this study are included in the article/Supplementary Material. Further inquiries can be directed to the corresponding authors.

## Supplementary Materials

The following supporting information can be downloaded at: https://www.mdpi.com/article/10.3390/insects17040378/s1, Table S1: Raw data of 129 investigation plots across four habitat types (farmlands, fishponds, orchards, and urban green spaces) in Guangzhou.

## Appendix A

**Table A1:** Occurrence level of RIFA across four habitat types (farmlands, fishponds, orchards, and urban green spaces) in Guangzhou.

Seasonality	Habitats	Sampling Area (mu/667 hm^2^)	Sampling Area (ha)	Number of Live Ant Nests	Average Density of Live Ant Nests (Nests/667 m^2^)	Average Density of Live Ant Nests (Nests/ha)	Average Occurrence Level
Spring	Urban Green Space	209.29	13.95	208	0.99	14.91	II
	Farmland	81.48	5.43	296	3.63	54.51	III
	Fishponds	76.67	5.11	186	2.43	36.40	II
	Orchard	43.2	2.88	129	2.99	44.79	II
	Total/Average	410.64	27.38	819	1.99	29.91	II
Summer	Urban Green Space	209.29	13.95	192	0.92	13.76	II
	Farmland	81.48	5.43	184	2.26	33.89	II
	Fishponds	76.67	5.11	113	1.47	22.11	II
	Orchard	43.2	2.88	77	1.78	26.74	II
	Total/Average	410.64	27.38	566	1.38	20.67	II
Autumn	Urban Green Space	209.29	13.95	310	1.48	22.22	II
	Farmland	81.48	5.43	245	3.01	45.12	II
	Fishponds	76.67	5.11	244	3.18	47.75	II
	Orchard	43.2	2.88	98	2.27	34.03	II
	Total/Average	410.64	27.38	897	2.18	32.76	II
Winter	Urban Green Space	209.29	13.95	115	0.55	8.24	I
	Farmland	81.48	5.43	215	2.64	39.59	II
	Fishponds	76.67	5.11	156	2.03	30.53	II
	Orchard	43.2	2.88	57	1.32	19.79	II
	Total/Average	410.64	27.38	543	1.32	19.83	II

Based on monthly dynamics in the number of active RIFA nests across the four habitats (Figure A1), the tea-oil camellia orchard supported the highest mean nest numbers, followed by fishponds, whereas the sugar apple and guava orchards generally exhibited lower nest numbers. Both the tea-oil camellia orchard and fishponds reached their annual peak in February, with mean values of 28.00 and 13.33 active nests, respectively. The sugar apple orchard peaked in January–February (7.33 nests), whereas the guava orchard peaked in June–July (7.66 nests). In fishponds, nest number in February was significantly higher than in July and August.

Regarding the monthly dynamic variation in nest volume (Table A2), mound volume in the tea-oil camellia orchard was significantly larger than that in fishpond habitats in May, August, October, and November (two-way ANOVA followed by Tukey’s multiple comparisons; Table A2). The tea-oil camellia orchard reached its annual maximum mound volume of 30,056.98 cm^3^ in May, whereas fishpond habitats peaked at 27,518.78 cm^3^ in April. Across months, both habitats showed higher mound volumes in March–May and October–December than in mid-summer months, indicating clear seasonal variation in mound volume within the two habitats.

**Table A2:** Monthly dynamic changes in the nest volume of RIFA in two habitats.

Month	Mound Height (cm)		Base Area of Mound (cm^2^)		Mound Volume (cm^3^)	
	Fishpond	Orchard Tea-Oil Camellia	Fishpond	Orchard Tea-Oil Camellia	Fishpond	Orchard Tea-Oil Camellia
1	11.80 ± 4.34	15.20 ± 3.82	1432.49 ± 738.65	928.50 ± 334.53	11,475.09 ± 8960.13 (Aa)	9630.50 ± 5098.39 (Aa)
2	18.90 ± 8.57	21.80 ± 3.68	1164.98 ± 532.40	962.27 ± 849.85	16,462.78 ± 13,435.25 (Aa)	14,608.30 ± 15,106.79 (Aac)
3	14.50 ± 4.95	19.10 ± 4.20	1008.37 ± 541.04	1060.29 ± 307.48	10,338.20 ± 9380.52 (Aa)	13,518.80 ± 5396.91 (Aad)
4	17.40 ± 6.54	24.70 ± 5.12	2307.34 ± 1114.35	1644.47 ± 711.54	27,518.78 ± 17,858.67 (Abc)	26,415.61 ± 11,575.38 (Abcde)
5	13.60 ± 2.76	24.20 ± 8.07	1657.98 ± 687.96	1776.96 ± 773.17	14,783.71 ± 7618.81 (Aac)	30,056.98 ± 20,686.69 (Bbcf)
6	8.90 ± 2.47	11.90 ± 5.93	1195.30 ± 558.95	1785.60 ± 671.23	7341.17 ± 4362.40 (Aa)	14,945.18 ± 10,578.31 (Aaefh)
7	11.30 ± 5.66	18.40 ± 6.24	1096.34 ± 916.11	1030.52 ± 465.15	6815.37 ± 5639.37 (Aa)	11,095.11 ± 2740.20 (Aae)
8	12.30 ± 6.29	15.10 ± 2.60	572.32 ± 406.67	1793.46 ± 367.69	4331.58 ± 2692.71 (Aa)	18,256.32 ± 5636.58 (Baefi)
9	12.70 ± 6.41	20.00 ± 5.56	702.62 ± 377.34	1160.98 ± 632.13	6710.49 ± 6100.25 (Aa)	15,143.37 ± 8821.98 (Aaefj)
10	12.50 ± 2.72	21.30 ± 4.16	479.56 ± 316.39	1109.85 ± 550.16	4033.54 ± 2802.15 (Aa)	15,478.63 ± 8104.35 (Baefk)
11	16.20 ± 4.96	22.00 ± 8.23	1356.93 ± 648.96	1734.16 ± 1087.22	15,833.57 ± 10,508.22 (Aac)	28,411.52 ± 22,625.79 (Bbcdhijkl)
12	12.60 ± 3.78	21.20 ± 6.63	1539.46 ± 730.02	1216.42 ± 645.31	13,394.23 ± 8632.99 (Aac)	18,142.75 ± 14,486.64 (Aaefl)

Note: Values in the table are presented as mean ± standard deviation (SD). Nest volume was analysed using two-way ANOVA with Month and Habitat as fixed factors, followed by Tukey’s multiple comparisons test. Uppercase letters indicate comparisons between habitats within the same month, and lowercase letters indicate comparisons among months within the same habitat; values sharing the same letter are not significantly different (*p* ≥ 0.05).
